# Gene Expression Profiling of Post Mortem Midbrain of Parkinson’s Disease Patients and Healthy Controls

**DOI:** 10.3390/ijms25020707

**Published:** 2024-01-05

**Authors:** Michele Salemi, Maria Ravo, Giuseppe Lanza, Francesca A. Schillaci, Giovanna Maria Ventola, Giovanna Marchese, Maria Grazia Salluzzo, Graziella Cappelletti, Raffaele Ferri

**Affiliations:** 1 Oasi Research Institute–IRCCS, 94018 Troin, Italy; glanza@oasi.en.it (G.L.); fran7.sch@gmail.com (F.A.S.); msalluzzo@oasi.en.it (M.G.S.); rferri@oasi.en.it (R.F.); 2 Genomix4Life Srl, 94081 Baroniss, Italy; maria.ravo@genomix4life.com (M.R.); giovanna.ventola@genomix4life.com (G.M.V.); giovanna.marchese@genomix4life.com (G.M.); 3 Genome Research Center for Health–CRGS, 94081 Baronissi, Italy; 4 Department of Surgery and Medical–Surgical Specialties, University of Catania, 95100 Catania, Italy; 5 Department of Biosciences, University of Milan, 20019 Milan, Italy; graziella.cappelletti@unimi.it

**Keywords:** mRNAs, RNA sequencing, Parkinson’s disease, transcriptome analysis, substantia nigra

## Abstract

Parkinson’s disease (PD) stands as the most prevalent degenerative movement disorder, marked by the degeneration of dopaminergic neurons in the substantia nigra of the midbrain. In this study, we conducted a transcriptome analysis utilizing post mortem mRNA extracted from the substantia nigra of both PD patients and healthy control (CTRL) individuals. Specifically, we acquired eight samples from individuals with PD and six samples from CTRL individuals, with no discernible pathology detected in the latter group. RNA sequencing was conducted using the TapeStation 4200 system from Agilent Technologies. A total of 16,148 transcripts were identified, with 92 mRNAs displaying differential expression between the PD and control groups. Specifically, 33 mRNAs were significantly up-regulated, while 59 mRNAs were down-regulated in PD compared to the controls. The identification of statistically significant signaling pathways, with an adjusted *p*-value threshold of 0.05, unveiled noteworthy insights. Specifically, the enriched categories included cardiac muscle contraction (involving genes such as ATPase Na^+^/K^+^ transporting subunit beta 2 (*ATP1B2*), solute carrier family 8 member A1 (*SLC8A1*), and cytochrome c oxidase subunit II (*COX2*)), GABAergic synapse (involving GABA type A receptor-associated protein-like 1 (*GABARAPL1*), G protein subunit beta 5 (*GNB5*), and solute carrier family 38 member 2 (*SLC38A2*), autophagy (involving *GABARAPL1* and tumor protein p53-inducible nuclear protein 2 (*TP53INP2*)), and Fc gamma receptor (FcγR) mediated phagocytosis (involving amphiphysin (*AMPH*)). These findings uncover new pathophysiological dimensions underlying PD, implicating genes associated with heart muscle contraction. This knowledge enhances diagnostic accuracy and contributes to the advancement of targeted therapies.

## 1. Introduction

PD stands out as the most prevalent movement disorder and neurodegenerative disease after Alzheimer’s dementia, affecting approximately seven million people globally [1,2]. Clinically, PD is a heterogeneous condition primarily characterized by a resting tremor, bradykinesia, and rigidity, commonly regarded as the “core motor symptoms”. However, PD is clinically diverse, presenting with various causes and clinical manifestations. While a resting tremor is often a prominent and traditionally associated sign of PD, its clinical variability can result in its absence in some cases.

Despite the multifaceted clinical presentation, current diagnosis remains primarily clinical, with laboratory tests, such as genetic testing, and instrumental exams, including structural and/or functional neuroimaging, reserved for patients with atypical presentations [3]. Diagnostic criteria define PD as the presence of bradykinesia combined with either a rest tremor, rigidity, or both [4]. However, the clinical spectrum encompasses additional motor symptoms and non-otor symptoms, some of which precede motor manifestations and are equally debilitating [5,6,7,8].

While non-specific symptoms, such as constipation, apathy, fatigue, or mild cognitive changes, are challenging to promptly link to PD onset or its preclinical/prodromal phase, others, like hyposmia, adult onset depression/anxiety, or REM sleep behavior disorder, are considered strong predictors of neurodegeneration. These symptoms play a crucial role in the early diagnosis of PD, even in the absence of common motor symptoms [9].

Among synucleinopathies, multiple system atrophy (MSA) and dementia with Lewy bodies (DLB) represent significant differential diagnoses. MSA is a sporadic neurodegenerative disease clinically characterized by parkinsonism or cerebellar ataxia, both combined with dysautonomia and a poor response to levodopa. Magnetic resonance imaging may reveal specific abnormalities, such as the “hot cross bun” sign and bilateral putaminal hyperintensity [10]. DLB is characterized by dementia preceding or developing alongside parkinsonism, with core features including fluctuating cognition, recurrent visual hallucinations, dysautonomia, and marked sensitivity to D2 receptor-blocking agents. Functional studies may show typical hypometabolism within the occipital cortex [11].

A comprehensive brain transcriptome study of subjects with MSA revealed the down-regulation of oligodendrocyte genes associated with a loss of myelination, particularly the *QKI* gene as a master regulator of this gene network. Additionally, they demonstrated the up-regulation of monomeric α-synuclein gene expression in neurons [12].

Despite considerable research efforts, encompassing both preclinical and clinical studies, PD remains incurable. The progressive loss of dopaminergic (DA) neurons in the substantia nigra pars compacta (SNpc), a crucial part of the midbrain regulating movement tone and velocity, persists without effective intervention, and the complete set of pathomechanisms behind this degeneration remains incompletely elucidated [1,13]. Pathophysiologically, PD exhibits a multifactorial origin, involving intricate interactions between various genetic and environmental factors, a phenomenon observed in other complex diseases, such as tumors [14]. While some factors exert their influence on the elderly population in general, others appear to be more specifically associated with this disorder [15]. It is noteworthy that post mortem studies consistently report that over 60% of DA neurons in the SNpc are already degenerated when overt clinical signs manifest. This indicates that PD is neuropathologically evident long before its clinical onset, suggesting a period during which the human brain can compensate for dopaminergic loss until reaching a “clinical threshold” for PD [16,17,18].

Histological studies reveal that PD is characterized by the abnormal deposition of the insoluble protein *α*-synuclein, forming aggregates known as Lewy bodies [19]. These protein aggregates progressively accumulate throughout the brainstem and various neocortical and limbic regions, reflecting the progressive degeneration of the entire central nervous system (CNS) [20]. More recently, a neuroinflammatory state has been observed in the brains of PD patients, particularly evident in the SNpc [21,22]. Both neuroinflammation and dysfunctional activation of the immune system within the CNS significantly contribute to PD pathology and pathophysiology [23]. The inflammasome, a crucial complex of immune-modulating receptors and sensors, plays a role in recruiting proteins associated with apoptotic mechanisms through caspase-1 activation [24,25]. Caspase-1, in turn, activates the proinflammatory cytokines interleukin (IL)-1β and IL-18, perpetuating the neuroinflammatory state in PD brains [25,26,27,28,29]. A recently proposed model suggests that alpha-synuclein activates the inflammasome in the SNpc, leading to IL-activated proinflammatory profiles, neuronal death, and clinical symptoms [30,31,32].

Genetically, several genes or gene variants, including leucine-rich repeat kinase 2 (*LRRK2*), synuclein alpha (*SNCA*), glucosylceramidase beta-1 (*GBA1*), Parkin RBR E3 ubiquitin protein ligase (*PARKIN*), and PTEN-induced kinase 1 (*PINK1*), have been implicated in causing PD [33,34,35,36]. Molecular profiling studies of post mortem SNpc samples, aimed at identifying differential molecular expression changes specific to PD compared to controls, have been conducted [37]. For instance, Simunovic et al. [38] used RNA microarrays to analyze SNpc gene expression in PD samples, identifying the dysregulation of known molecular regulatory pathways in PD, including dysfunction in mitochondrial and oxidative-stress-induced cellular responses [39,40]. In a recent comparative gene expression analysis on laser-dissected neurons from SNpc, Zaccaria et al. [41] revealed 52 dysregulated genes in PD samples compared to controls.

We recognize that synucleinopathies are neurodegenerative disorders associated with the misfolding and aggregation of Alpha-synuclein (*α-Syn*) [42]. Real-time tremor-induced conversion technology (RT-QuIC) is an in vitro amplification method initially developed for the analysis of the prion protein (PrP) [42]. Today, it is also employed for the biochemical assessment of different strains of *α-Syn* within synucleinopathies [42].

Although PD is encompassed within this family of pathologies (synucleinopathies), our study focused on transcriptome analysis by examining post mortem mRNA. This approach allows the targeted identification of gene expression, a critical condition for comprehending this well-known yet enigmatic pathology present in the global population. Transcriptomics enables the unveiling of microscopic changes, offering the potential to identify individuals at risk of developing the disease, even in the presence of non-motor symptoms, or to anticipate the evolution of the pathology.

The review by Rike and Stern [43] cites proteomic and transcriptomic studies involving post mortem brain samples from individuals with PD compared to controls. Some authors of these studies concentrated on the transcriptome in the substantia nigra [44,45]. The data obtained revealed that the dysregulated groups of genes/pathways/biological processes in individuals with PD, as opposed to controls, are more associated with extracellular matrix (ECM)-receptor interaction and cell adhesion molecules.

In our study, we conducted mRNA analysis and subsequent enrichment using the Kyoto Encyclopedia of Genes and Genomes (KEGG) and Gene Ontology (GO) to assess mRNAs extracted post mortem from the SN of subjects with PD and healthy controls. Utilizing samples obtained from Parkinson’s UK Brain Bank (Imperial College London, London, UK), we specifically addressed a case history of PD at Braak LB Stage 6.

## 2. Results

**Figure 1:**
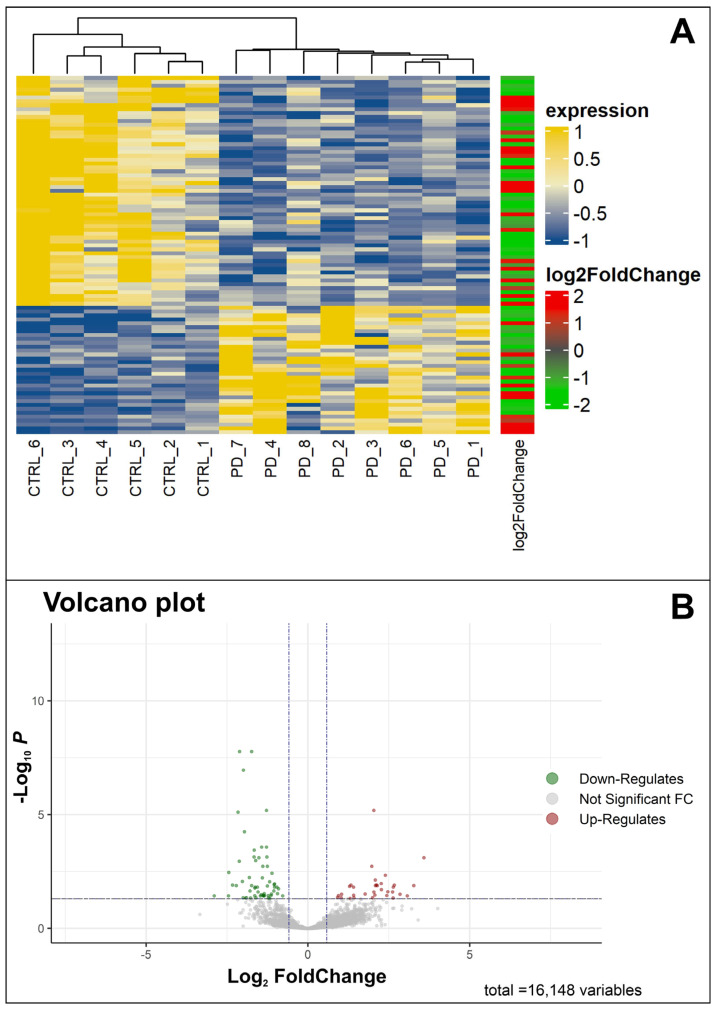
Visualization of differentially expressed genes (DEGs). (**A**) Heatmap of significant DEGs in patients with Parkinson’s disease (PD) and healthy control (CTRL) individuals. In yellow, we see the genes with an up-normalized expression level, whereas in blue, we see the down genes. The log2 (foldChange) bar indicates, in red and in green, the up- and down-regulated genes, respectively. (**B**) Volcano plot of significant DEGs based on fold changes and *p*-values. The green color shows the down-regulated genes, whereas the red color shows the up-regulated genes.

In the examination of mRNA deregulation in the SNpc of PD patients, we conducted gene expression profiling on eight post mortem SNpc samples from PD patients and six from healthy CTRL subjects using next-generation sequencing (RNA-Seq). The aim was to identify specific and differential changes in molecular expression. Following the removal of low-quality reads and adapter sequences, the high-quality reads were aligned against the human genome reference (hg38). In detail, we identified a total of 16,148 transcripts (Supplementary Table S1, sheet A), with 92 mRNAs differentially expressed (DEGs) between the two groups (PD vs. CTRL). Among these, 33 mRNAs were significantly up-regulated (Table 1, Figure 1), while 59 mRNAs were significantly down-regulated in PD compared to CTRL (Table 2, Figure 1). The normalized count of mRNAs is available at ArrayExpress (E-MTAB-13295).

The heatmap (Figure 1A) illustrates statistically significant differences in mRNA expression profiles between PD and CTRL. The volcano plot (Figure 1B) depicts the distribution of differentially expressed transcripts by their fold change and *p*-values. The most-up-regulated genes are toward the right, the most-down-regulated are toward the left, and the most statistically significant genes are at the top.

**Figure 2:**
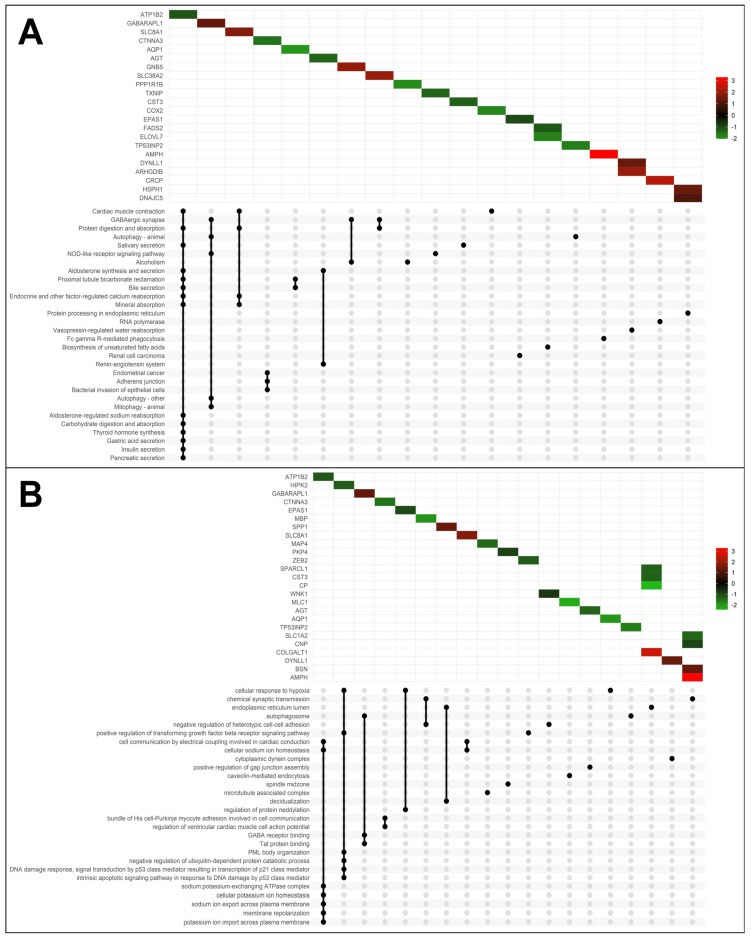
KEGG and GO enrichment analysis of differentially expressed genes. (**A**) UpSet Plot shows the intersections of significant genes and top 30 enriched KEGG pathway and (**B**) GO terms, with the Log2FoldChange scale.

We employed the pathfindR tool to analyze significant DEGs in PD based on the KEGG pathway database. Enrichment analysis was performed to explore functional variations between the two groups and investigate pathways potentially associated with PD. Statistically significant signaling pathways included (hsa04260) cardiac muscle contraction, (hsa04727) GABAergic synapse, (hsa04140) autophagy, and (hsa04666) Fc gamma R-mediated phagocytosis (Figure 2A and Supplementary Table S1).

For GO enrichment, we identified both down-regulated and up-regulated genes involved in various molecular functions or biological processes (Figure 2B and Supplementary Table S2). Interestingly, among them, we found the enrichment of (GO:0007268) chemical synaptic transmission (*AMPH*, bassoon presynaptic cytomatrix protein (*BSN*), 2′,3′-cyclic nucleotide 3′ phosphodiesterase (*CNP*), myelin basic protein (MBP), solute carrier family 1 member 2 (*SLC1A2*), (GO:0071456) cellular response to hypoxia (aquaporin 1 (*AQP1*), endothelial PAS domain protein 1 (*EPAS1*), homeodomain-interacting protein kinase 2 (*HIPK2*), (GO:0000045) autophagosome assembly (*GABARAPL1, TP53INP2*), (GO:0050811) GABA type A receptor binding (*GABARAPL1*), and (GO:0086064) cell communication via electrical coupling involved in cardiac conduction (*SLC8A1, ATP1B2*).

**Figure 3:**
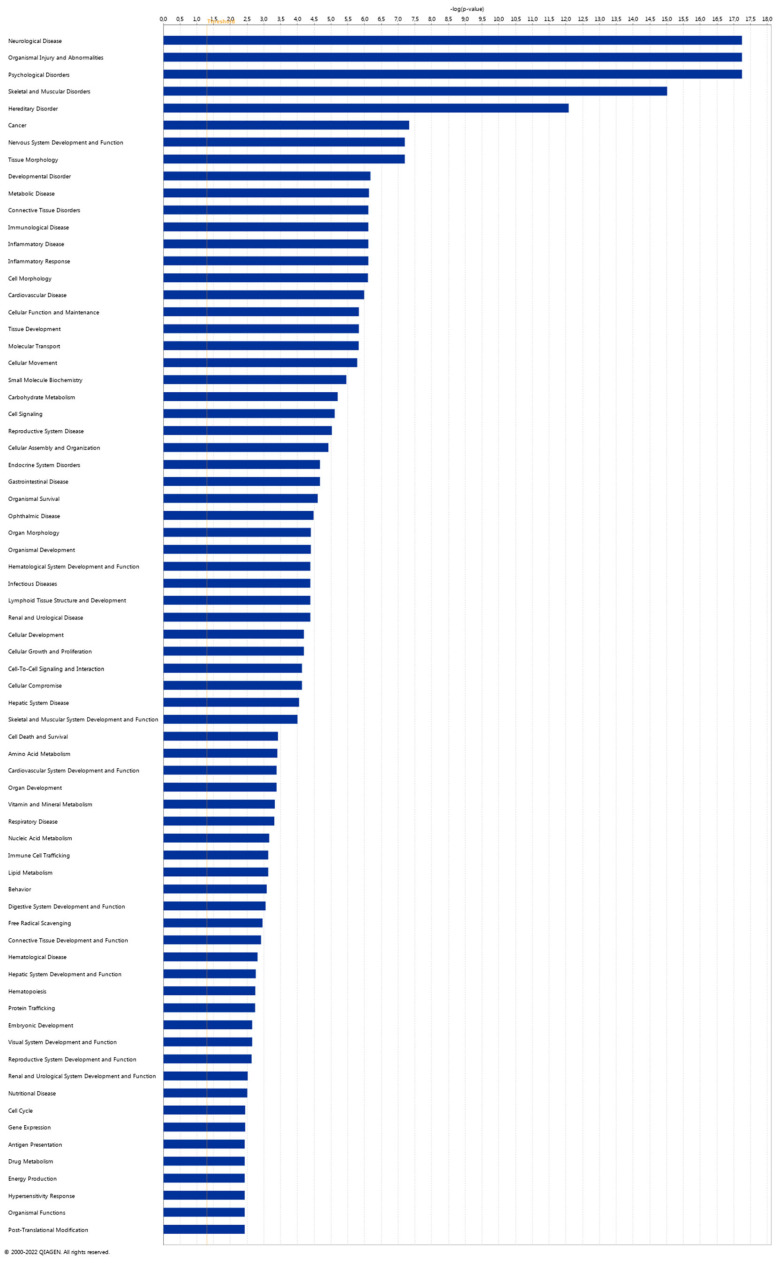
Ingenuity Pathway Analysis (IPA). Disease and Function analysis of differentially expressed genes in the IPA software (Prism version 9.4.1, 2022) with Log2 (*p*-value) scale.

**Figure 4:**
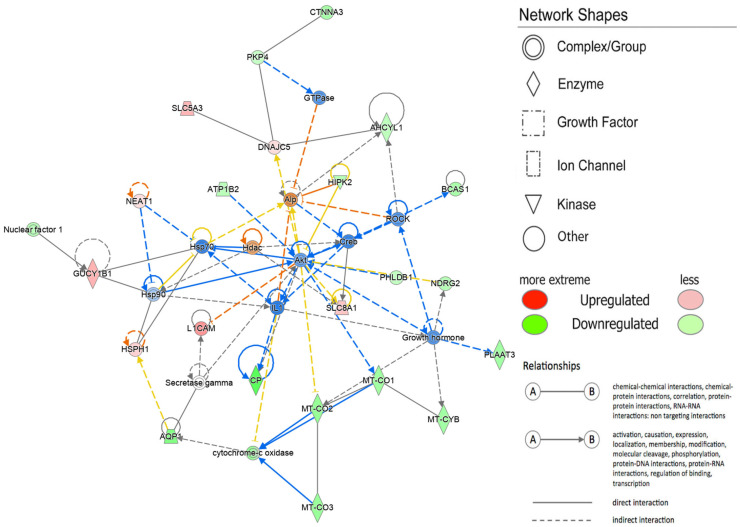
Network by Ingenuity Pathway Analysis (IPA). Neurological Disease, Organismal Injury and Abnormalities, Psychological Disorders. Red indicates the up-regulated transcripts, whereas green indicates the down-regulated transcripts. The color of the lines indicates the different relationship states. In particular, the orange lines show an activation, the blue lines show an inhibition, gray indicates non-predicted effects, and yellow indicates findings inconsistent with the state of the downstream molecule.

To explore the Diseases and Biological Functions significantly enriched in DEGs and assess potential associations with PD susceptibility in the SNpc, we employed Ingenuity Pathway Analysis (IPA). The analysis revealed significant enrichment in Neurological Disease, with multiple annotations related to disorders of the basal ganglia, movement disorders, neuromuscular disease, dyskinesia, progressive motor neuropathy, familial neurological disorders, Parkinson’s disease, progressive neurological disorders, abnormal morphology of the nervous system, and tauopathy (Figure 3 and Supplementary Table S3). The DEG IPA Network Analysis identified seven networks with nodes and interactions associated with the top Diseases or Function Annotations, such as Neurological Disease, Organismal Injury and Abnormalities, and Psychological Disorders (Figure 4 and Table 3).

## 3. Discussion

While PD was initially described by James Parkinson in 1817, it remains a disease with several knowledge gaps, spanning the ethnic divide, gender differences, and the field of PD genetics [46]. Numerous studies in the field of omics sciences (genomics, transcriptomics, proteomics, metabolomics) have contributed to the identification of new genes, pathways, and proteins, providing insight into the extensive variability of PD in the context of current trends in personalized medicine [47].

Referring to the review by Rike and Stern [43], our study, at the pathway analysis level, reveals some overlaps with interaction mechanisms and cell adhesion molecules. Importantly, we have identified data that were not previously highlighted in the literature.

The results obtained indicate 33 significantly up-regulated mRNAs (padj ≤ 0.05 and |FC| ≥ 1.5) and 59 significantly down-regulated mRNAs (padj ≤ 0.05 and |FC| ≤ −1.5) in PD subjects compared with CTRLs.

Among these, the genes most involved in the highlighted pathways, functions, and networks include *ATP1B2, GABARAPL, SLC38A*, mitochondrially encoded cytochrome c oxidase I (*MT-CO1*), mitochondrially encoded cytochrome c oxidase II (*MT-CO2*), mitochondrially encoded cytochrome c oxidase III (*MT-CO3*), mitochondrially encoded cytochrome b (*MT-CYB*), and cytochrome oxidase.

*ATP1B2*, a plasma membrane pump with diverse functions, including homeostasis in cell differentiation and apoptosis, is particularly expressed in the brain [48]. In our study, the *ATP1B2* gene was under-expressed in PD brains compared to CTRLs (Table 1), confirming its peculiar expression in normal brains, as previously reported [49]. Following KEGG and GO enrichment analysis, *ATP1B2* correlated with most of the relevant pathways observed (Figure 2A,B). Notably, in KEGG analysis, one pathway is “contraction of cardiac muscles”, while in GO analysis, *ATP1B2* correlates with the “cellular communication via electrical coupling involved in cardiac conduction” pathway (Figure 2A,B).

The cardiac sodium/calcium exchanger (*SLC8A1*), found to be overexpressed (Table 2) in our data, is a bidirectional calcium transporter contributing to the electrical activity of the heart. It is abundantly expressed in the heart and less so in the brain, retina, and skeletal muscle [50,51]. Enriched categories included different genes involved in cardiac muscle contraction, highlighting the known clinical and experimental evidence linking cardiac changes and PD. Although most dysautonomic symptoms in PD arise from alterations in the peripheral nerves of the autonomic nervous system [52], a direct role of myocardial cell pathology in PD patients cannot be excluded.

Recent evidence shows a close link between PD and cardiac cell dysfunction, with mitophagy likely playing a crucial role [53,54]. Proteins like those encoded by the *ATP1B2* gene, integral membrane proteins responsible for establishing and maintaining electrochemical gradients, may connect cardiac dysfunction with the PD patients studied here [55].

Notably, Bardutz et al. [56] recently suggested alterations in systolic function in PD patients, possibly related to dysfunctional cardiac muscle contraction. Oleksakova et al. [57] highlighted differences in baroreflex function and the baroreflex-mediated vasoconstriction response to orthostasis in PD patients. While data in the literature associate cardiac dysfunction with PD, our study directly underscores the dysregulation of genes related to cardiac contraction in the brain of PD patients. The specific role of these dysregulated genes in brain function remains unclear, but we cannot exclude their potential contribution to the neurodegeneration observed in PD.

Among the genes of interest, *GABARAPL1* was found to be overexpressed, as indicated via both KEGG and GO analyses, involving a distinct set of pathways (five and three, respectively): GABAergic synapse, autophagy—animal, the nucleotide-oligomerization-domain (NOD)-like receptor signaling pathway (a specialized group of intracellular proteins that plays a critical role in the regulation of the host innate immune response), autophagy—other, and mitophagy—animal (according to KEGG), as well as autophagosome, GABA type A receptor binding, and Tat protein binding (according to GO). The Tat protein serves as a nuclear transactive regulator of viral gene expression, either for human immunodeficiency virus (HIV) or the equivalent protein of another virus (https://www.uniprot.org/uniprotkb/P04610/entry; accessed on 10 December 2023). Nagel et al. [58] demonstrated that the Tat-heat shock protein 70 (Hsp70) complex effectively prevents neuronal cell death in both in vitro and in vivo models of PD.

Autophagy, a highly conserved cellular degradation process regulated by specific autophagy-related (Atg) factors, entails the formation of double-membrane autophagosomes that engulf cytoplasmic components for degradation. In mammals, this process is complex due to the presence of six Atg8 homologues, categorized into the GABA type A receptor-associated protein (*GABARAP*) and microtubule-associated protein 1 light-chain 3 (*MAP1LC3*) subfamilies [59]. *GABARAPL1/GEC1*, a member of the GABARAP subfamily, exhibits the highest mRNA expression among Atg8 homologues in the CNS. Notably, *GABARAPL1* brain expression is observable as early as embryonic day 11, increasing progressively to peak in adulthood. Significantly, *GABARAPL1* expression in the adult brain is particularly intense in neurons involved in motor and neuroendocrine functions, notably in the SNpc [60]. A dysregulation of Atg8 homologues has been observed in other synucleinopathies, such as Lewy-body dementia and multiple-system atrophy [61]. In PD, alterations in autophagic mechanisms are evident, as demonstrated by transcript levels of several autophagy genes in blood cells. A recent study found the overexpression of autophagy-related genes, including *MAP1LC3B*, *GABARAP*, *GABARAPL1*, *GABARAPL2*, and sequestosome 1 (*P62/SQSTM1*), in PD patients, with potential implications for predicting markers and therapeutic responses [62].

Among the highlighted genes, *AMPH* stands out, as it was found to be overexpressed, as indicated via both the KEGG and GO analyses. This overexpression involves pathways such as phagocytosis mediated by Fc gamma R (according to KEGG) and chemical synaptic transmission (according to GO). The crystallizable fragment receptor (FcR) is a receptor with a specific binding capacity for the Fc region of the antibody tail and is responsible for inducing phagocytosis. When Fc gamma R on monocyte-macrophages or neutrophils combines with IgG through its Fc region, it triggers phagocytosis [63,64]. FcRs play a crucial role in normal responses to infection or tissue injury and are implicated in immune-related diseases and increasingly observed in neurodegenerative diseases [65]. The aberrant activation of FcRs in neural cells may contribute to the pathogenesis of major neurodegenerative conditions, including Alzheimer’s disease, Parkinson’s disease, ischemic stroke, and multiple sclerosis [65]. This observation aligns with the pathway expression found in our study sample.

Similarly, the *SLC38A2* gene was identified as overexpressed and associated with two relevant pathways according to KEGG: GABAergic synapse and protein digestion and absorption. This finding strengthens the connection between GABA overexpression, PD pathology, and neural degeneration. In a recent study using a rotenone-induced PD rat model [66], nardosinone, a biochemical compound enhancing NGF-mediated neurite outgrowth and synaptogenesis, demonstrated anti-PD efficacy. Transcriptome and proteome analyses suggested that the anti-PD target of nardosinone is the *SLC38A2* gene, potentially involving the GABAergic synaptic pathway. This underscores the *SLC38A2* gene as a potential target for PD treatment and emphasizes its modulatory effects as an anti-PD agent through the GABA system [66]. Diseases and Function analysis in the IPA software highlighted that DEGs correlated with Neurological Disease (Figure 3 and Figure 4). Network IPA Analysis (Figure 4) revealed the down-regulation of many mitochondrial genes in the SN of PD subjects, including *MT*-*CO1*, *MT*-*CO2*, *MT*-*CO3*, *MT*-*CYB*, and cytochrome oxidase. This further supports mitochondrial dysfunction in PD, where mitochondria, involved in crucial functions, primarily energy generation, are essential for nearly all cellular activities. Alterations in mitochondrial functioning lead to insufficient energy production, particularly affecting the CNS [67]. Evidence indicates that mitochondrial respiratory-chain dysfunction plays a primary role in various neurodegenerative diseases, including PD [67,68]. Impaired elements of the respiratory chain, such as defects in complex I, have been associated with PD and frontal cortex dysfunction [69,70,71,72]. Damage to the electron transport chain increases oxidative stress and neuronal dysfunction, potentially contributing to the onset and progression of PD. Progressive mitochondrial damage results in the accumulation of non-functional mitochondria, further contributing to neuronal degeneration [73].

Looking at the down-regulated genes in the SN of PD patients, a group of genes involved in maintaining the structure and function of glial cells was noted. These genes include *MBP, CPN* (myelin protein cyclic nucleotide phosphodiesterase)*, CTNNA3* (alpha-T-catenin), *AQP1*, and glial fibrillary acidic protein (*GFAP*). Among the up-regulated genes, *SLC8A1* is involved in linking trans-plasmalemmal gradients of sodium and calcium ions to the membrane potential of astrocytes. This outcome suggests the emerging important role of glial cells in neurodegeneration and PD pathogenesis [74,75,76,77,78,79].

## 4. Materials and Methods

### 4.1. Human Post Mortem Midbrain Samples

Human midbrain samples were generously provided by the Parkinson’s UK Brain Bank (Imperial College London, London, UK). A total of 8 PD (6 males and 2 females; mean age 81.125 and SD of 5.693) and 6 CTRL (1 male and 5 females; mean age 78.166 and SD of 10.381) samples were acquired, and the specimens were histologically sectioned at the midbrain, encompassing the human SN in all slides. Each section had a thickness of 4 µm. Supplementary Table S4 presents a concise summary of the clinical characteristics of PD subjects and controls, with the data sourced from the Parkinson’s UK Brain Bank (Imperial College London, London, UK). It is noteworthy that all PD cases included in the study are classified at Braak LB Stage 6. The study adhered to the principles of the Declaration of Helsinki of 1964 and its subsequent amendments. The Ethics Committee of the Oasi Research Institute—IRCCS of Troina (Italy) granted approval for the protocol on 5 April 2022 (approval code: 2022/04/05/CE-IRCCS-OASI/52).

### 4.2. RNA Isolation from Human Midbrain Samples

RNA was extracted from 4 µm of Formalin-fixed paraffin-embedded (FFPE) slide-mounted sections using the RecoverAll Total Nucleic Acid Isolation Protocol (Thermo Fisher Scientific Inc., Waltham, MA, USA), following the manufacturer’s instructions. Subsequently, the RNA was stored at −80 °C until further processing.

### 4.3. RNA Sequencing and Functional Analysis

RNA sequencing and subsequent data analysis were conducted by Genomix4Life Srl (Baronissi, Italy). The quality and quantity of RNA were assessed using a Qubit fluorometer (Thermo Fisher Scientific Inc.) and a TapeStation 4200 (Agilent Technologies, 5301 Stevens Creek Blvd), respectively.

Indexed libraries were prepared from 50 ng of purified RNA each, employing the Illumina Stranded Total RNA with Ribo-Zero Plus Kit (Illumina Inc., San Diego, CA, USA), following the manufacturer’s guidelines. Library quantification was performed using the TapeStation 4200 (Agilent Technologies, Santa Clara, CA, USA) and Qubit. Subsequently, the indexed libraries were pooled in equimolar amounts, resulting in a final concentration of 2 nM. The Illumina NovaSeq 6000 System was utilized to sequence the pooled samples in a 2 × 75 paired-end format.

Trimming of short reads (<25 bp) and removal of adapter sequences were carried out with cutadapt (v.2.8) [80]. Using STAR [81] software (version 2.7.3a), the trimmed fastq files were mapped to the reference genome (GenCode (HG38-Release 37 (GRCh38.p13)). [https://www.gencodegenes.org/human/ accessed on 1 August 2023]) STAR [81] was used with standard parameters to create paired-end fastq files and wrote several output files, such as alignments (SAM/BAM). The next step of RNA-seq workflow, involved the gene quantification per sample, was performed using the featureCounts (version 2.0) tool. FeatureCounts is a highly efficient read summarization program that counts mapped reads (SAM/BAM files) for genomic features such as genes [https://bioweb.pasteur.fr/docs/modules/subread/1.4.6-p3/SubreadUsersGuide, accessed on 29 December 2023]. A Custom R script was employed for data normalization and differential expression analysis using the Bioconductor DESeq2 [82] package, based on negative binomial generalized linear models and on the estimates of dispersion and logarithmic fold changes incorporated in data-driven prior distributions. The threshold for considering genes as differentially expressed was set at Fold-Change ≥ 1.50 or ≤−1.50 (|FC| ≥ 1.50) with adjusted *p*-values ≤ 0.05 (padj). Volcano plots and heatmaps were generated using the En-hancedVolcano (10.18129/B9.bioc.EnhancedVolcano) and ComplexHeatmap [83] packages in R.

For functional analysis, KEGG pathway and GO database analyses were conducted using the R package pathfinder. Additionally, to gain a deeper understanding of the complex transcriptomics data, Ingenuity Pathway Analysis (IPA) (Prism version 9.4.1, 2022) [84] was performed, specifically for investigating diseases and function analysis.

The raw data (.fastq files) and the normalized count of identified mRNAs are available on ArrayExpress (E-MTAB-13295).

## 5. Conclusions

One of the study’s limitations is undoubtably related to the number of cases examined, which stems from the necessity to select samples with a high yield of extracted mRNA. A future objective includes expanding the sample size of PD subjects to enhance clinical heterogeneity and explore the pathological anatomy further. Another limitation is the absence of an expression study on non-coding RNAs (ncRNAs), which could be a focus of subsequent research.

The in-depth analysis conducted on the transcriptome of our samples revealed statistically significant findings pertaining to various signaling pathways, including cardiac muscle contraction, GABAergic synapse, autophagy, the Fc gamma R-mediated phagocytosis signaling pathway, the cellular response to chemical synaptic transmission, hypoxia, autophagosome assembly, GABA type A receptor binding, and cellular communication via electrical coupling involved in cardiac conduction. These results were obtained through KEGG and GO enrichment analyses. Notably, we highlight gene expression alterations affecting “cardiac muscle contraction” and “cellular communication via electrical coupling involved in cardiac conduction” in PD brain tissues compared to CTRLs. This leads us to speculate that genes conventionally associated with electrical conduction mechanisms in cardiac muscle might also play a role in the brain, with their function still unclear. We cannot exclude the involvement of differentially expressed genes, such as *SLC8A1* and *ATP1B2*, in various tissues within the context of PD.

Another significant finding is the altered expression of mitochondrial genes in the SN of subjects with PD. While this has been discussed in the literature, our study reaffirms it, emphasizing the central role of mitochondrial genes in PD. These discoveries may pave the way for improved diagnostic precision and the development of novel targeted therapies.

## Figures and Tables

**Table 1:** mRNAs down-expressed in PD subjects compared to controls (padj ≤ 0.05 and |FC| ≥ 1.5).

Gene ID	Fold Change	Gene ID	Fold Change
*ETNPPL*	−7.435	*AC093330.1*	−2.718
*MTND4P12*	−5.475	*MAP4*	−2.693
*CP*	−5.444	*SLC1A2*	−2.651
*MLC1*	−5.02	*TXNIP*	−2.625
*PPDPF*	−4.631	*AGT*	−2.61
*PAQR6*	−4.469	*RHOBTB3*	−2.604
*ACBD7*	−4.345	*CST3*	−2.562
*MOBP*	−4.315	*SPARCL1*	−2.55
*AQP1*	−4.069	*MTND2P28*	−2.544
*TAGLN*	−4.001	*HIPK2*	−2.427
*MBP*	−3.977	*ZEB2*	−2.425
*PAIP2B*	−3.892	*NDRG2*	−2.408
*PPP1R1B*	−3.766	*MTURN*	−2.382
*MTATP6P1*	−3.736	*DAAM2*	−2.381
*MT-C02*	−3.5	*NFIX*	−2.343
*ELOVL7*	−3.456	*FADS2*	−2.325
*SCARA3*	−3.419	*ATP1B2*	−2.268
*MT-C03*	−3.338	*HEPACAM*	−2.263
*TP53INP2*	−3.336	*SHTN1*	−2.203
*FAM107A*	−3.168	*FAR1*	−2.182
*SEPTIN4*	−3.158	*AHCYL1*	−2.157
*ALAD*	−3.122	*EPAS1*	−2.075
*MT-CO1*	−3.116	*PHLDB1*	−2.058
*IRAG1*	−3.089	*GFAP*	−2.043
*PLAAT3*	−3.056	*MAP4K4*	−2.041
*MT-CYB*	−3.045	*PADI2*	−1.938
*CTNNA3*	−2.949	*CNP*	−1.916
*FAT3*	−2.906	*PKP4*	−1.874
*BCAS1*	−2.857	*WNK1*	−1.714
*TSC22D4*	−2.767		

**Table 2:** mRNAs over-expressed in PD subjects compared to controls (padj ≤ 0.05 and |FC| ≥ 1.5).

Gene ID	Fold Change	Gene ID	Fold Change
*IL10RA*	12.026	*FYB1*	4.204
*AMPH*	9.664	*ARHGDIB*	4.111
*HS6ST3*	8.405	*SLC38A2*	4.104
*VSNL1*	7.195	*GNB5*	3.998
*COLGALT1*	6.373	*NAA30*	3.930
*PRDM11*	6.234	*SLC8A1*	3.409
*L1CAM*	6.208	*DYNLL1*	2.680
*GPR34*	6.141	*HSPH1*	2.666
*ZNF618*	5.543	*SPP1*	2.660
*RCSD1*	5.419	*BSN*	2.518
*CRCP*	5.257	*GABARAPL1*	2.471
*INA*	4.831	*YWHAG*	2.443
*PTPRT*	4.812	*QDPR*	2.060
*GUCY1B1*	4.422	*DNAJC5*	2.042
*SLC5A3*	4.338	*NEAT1*	1.926
*SRGN*	4.250	*TNPO1*	1.890
*SCN8A*	4.236		

**Table 3:** Molecule networks obtained via Ingenuity Pathway Analysis (IPA).

Molecules in Network	Score	Focus Molecules	Diseases and Functions
*AHCYL1*, Akt, Alp, *AQP1*, *ATP1B2*, *BCAS1*, *CP*, Creb, *CTNNA3*, cytochrome-c oxidase, *DNAJCS*, growth hormone, GTPase, *GUCY1B1*, Hdac, *HIPK2*, Hsp70, Hsp90, *HSPH1*, *IL1*, *L1CAM*, *MT-C01*, *MTC02*, *MT-C03*, *MT-CYB*, *NDRG2*, *NEAT1*, nuclear factor M1, *PHLDB1*, *PKP4*, *PLAAT3*, *ROCK*, secretase gamma, *SLCSA3*, *SLC8A1*	49	22	Neurological Disease, Organismal Injury and Abnormalities, Psychological Disorders
14-3-3, 20s proteasome, 26s Pro teasome, *ALAD*, *BSN*, calmodulin, calpain, *CG*, *CNP*, *COLGALT1*, collagen Alpha1, collagen type I (complex), collagen type IV, *EPAS1*, *ERK1/2*, *FAR1*, focal adhesion kinase, *GFAP*, *HEPACAM*, *INA*, insulin, *MAP4*, *MBP*, *MLC1*, *PDGFBB*, Pka, *PP2A*, *SEPTIN4*, *SLC1A2*, *SRGN*, *TAGLN*, Tgf beta, transglutaminase, *VSNL1*, *WNK1*	38	18	Cellular Function and Maintenance, Nervous System Development and Function, Tissue Development
*AGT*, *AMPH*, Ap1, *ARHGDIB*, calcineurin protein(s), CD3, collagen type I (family), cytokine, *ELOVL7*, *FYB1*, *GNBS*, Gsk3, IKK (complex), IL12 (complex), integrin, integrin alpha L beta 2, Jnk, *LDL*, *MAP4K4*, Mek, *MTURN*, NFAT (complex), Nfat (family), *NFIX*, NFkB (complex), Nrlh, P38 MAPK, *PAD12*, Pkc(s), *PPP1R1B*, Rac, *SPARCL1*, *SPP1*, TCR, voltage-gated calcium channel Act in, *AMPK*, Ck2, *CLEC9A*, *CST3*, *DYNLL1*	25	13	Cardiovascular Disease, Cell-To-Cell Signaling and Interaction, Organismal Injury and Abnormalities
Actin, *AMPK*, Ck2, *CLEC9A*, *CST3*, *DYNLL1*, *ERK*, F Actin, *FADS2*, FAM 107 A, *GABARAPL1*, *GPR34*, hemoglobin, histone h3, histone h4, IgG, *IL10RA*, IL12 (family), immunoglobulin, interferon alpha, Mapk, MHC class II (complex), Notch, P13K (complex), RNA polymerase 11, *SHTN1*, Siglech, SRC (family), trypsin, tubulin, *TXNIP*, ubiquitin, Vegf, *YWHAG*, *ZEB2*	20	11	Cell-To-Cell Signaling and Interaction, Infectious Diseases, Organismal Injury and Abnormalities
*ACOD1*, *CARD16*, *CASP8*, Cd24a, *COL2A1*, cytokine receptor, D-glucose, *DAAM2*, *FAT3*, *GBPS*, *HCAR2*, *HEPACAM2*, *IFNG*, ligp1, *IL10RB*, *IL17RE*, *IL18BP*, *IL2RA*, *IRAGl*, *LGALS1*, *LTC4S*, *MLKL*, *NAA30*, *NLRCS*, *PARVG*, *PLAAT3*, *PRDM11*, *QDPR*, *REL*, *RHOBTB3*, *SCARA3*, Tlr11, *TNFRSF10B*, *TP531NP2*, *ZBP1*	18	10	Gastrointestinal Disease, Inflammatory Response, Organismal Injury and Abnormalities
*CFB*, *CHADL*, *CNTLN*, *CSNK1A1*, *EP300*, *ETNPPL*, *FAM110D*, *FAM83G*, *FRMD4A*, *FRY*, *HDAC4*, *HDAC5*, *IKZF2*, *IL15RA*, importin alpha, *MECOM*, miR-129-Sp (and other miRNAs w/seed UUUUUGC), *MOBP*, *NRBP2*, *PAIP2B*, *PAQR6*, *PDCD1LG2*, *PPDPF*, *PRMT1*, *RCSD1*, *SCN8A*, *SMARCB1*, *SNX22*, *SNX24*, *SOX2*, *SOX9*, *TNP01*, *TSC22D4*, *ZDBF2*, *ZNF618*	18	10	Carbohydrate Metabolism, Cell Cycle, Cellular Assembly and Organization
*ACBD7*, betaestradiol, *CA2*, *CALCRL*, clathrin, *CRCP*, *DEFB116*, *DNPH1*, *DOCK3*, *ERBB*, *FMOS*, *HS6ST3*, *HTR4*, *INPP5F*, L-histidine, *L1CAM*, Ly6a (includes others), *MAL*, *NOS1*, *OGDHL*, *OGN*, Pplc, *PROTEASE*, *PTEN*, *PTPRT*, *PYGL*, *SEMA3A*, sGC, *SLC38A2*, *SLC02B1*, *SRC*, sulfotransferase, *SULT1C2*, *TBC1D24*, Wap	10	6	Cellular Development, Connective Tissue Development and Function, Skeletal and Muscular System Development and Function

## Data Availability

https://www.bioinformatics.babraham.ac.uk/projects/fastqc/ accessed on 29 December 2023 (E-MTAB-13295).

## Supplementary Materials

The following supporting information can be downloaded at: https://www.mdpi.com/article/10.3390/ijms25020707/s1.
