# Supplementary material for: Gene Expression Profiling of Post Mortem Midbrain of Parkinson’s Disease Patients and Healthy Controls
Source: Int J Mol Sci. 2024 Jan 5;25(2):707. doi: 10.3390/ijms25020707 (PMC10815072; doi:10.3390/ijms25020707)
Supplement: Supplementary file 1 [file ijms-25-00707-s001.zip › Supplementari Table S4.pdf]

Supplementary Table S4. clinical features on the subjects included in the study.

Autopsy Id	Brain Bank	Age	Sex	Braak LB Stage	Braak Tangle	Clinical Diagnoses
PD1	Multiple Sclerosis and Parkinson's Tissue Bank	83	M	Parkinson's/Lewy body findings, Braak LB stage 6, Neocortical Lewy body disease	Braak tangle stage II	Parkinson disease (Present) [+G20] ons:74.00 dur:10.00,
PD2	Multiple Sclerosis and Parkinson's Tissue Bank	79	M	Parkinson's/Lewy body findings, Braak LB stage 6, Neocortical Lewy body disease	Braak tangle stage II	Parkinson disease (Present) [+G20], Multiple system atrophy, parkinsonian type [MSA-P] (Present) [+G23.2]
PD3	Multiple Sclerosis and Parkinson's Tissue Bank	76	F	Parkinson's/Lewy body findings, Braak LB stage 6, Amygdala-only Lewy body disease	Braak tangle stage II	Parkinson disease (Present) [+G20] ons:53.00 dur:22.00
CRTL1	Multiple Sclerosis and Parkinson's Tissue Bank	84	M			No abnormality detected (Present) [+NAD] ons:0.00 dur:0.00
CTRL2	Multiple Sclerosis and Parkinson's Tissue Bank	84	F			No abnormality detected (Present) [+NAD] ons:0.00 dur:0.00
CTRL5	Multiple Sclerosis and Parkinson's Tissue Bank	74	F			Multiple sclerosis (Present) [+G35], No abnormality detected (Present) [+NAD]
PD4	Multiple Sclerosis and Parkinson's Tissue Bank	89	F	Parkinson's/Lewy body findings, Braak LB stage 6, Limbic Lewy body disease	Braak tangle stage II	Parkinson disease (Present) [+G20] ons:65.00 dur:24.00
PD5	Multiple Sclerosis and Parkinson's Tissue Bank	80	M	Parkinson's/Lewy body findings, Braak LB stage 6, Neocortical Lewy body disease	Braak tangle stage II	Parkinson disease (Present) [+G20] ons:71.00 dur:8.00, Progressive supranuclear ophthalmoplegia [Steele-Richardson-Olszewski] (Present) [+G23.1]
CTRL6	Multiple Sclerosis and Parkinson's Tissue Bank	59	F			No abnormality detected (Present) [+NAD]
PD6	Multiple Sclerosis and Parkinson's Tissue Bank	89	M	Parkinson's/Lewy body findings, Braak LB stage 6, Neocortical Lewy body disease	Braak tangle stage II	Parkinson disease (Present) [+G20] ons:78.00 dur:11.00
PD7	Multiple Sclerosis and Parkinson's Tissue Bank	73	M	Parkinson's/Lewy body findings, Braak LB stage 6, Neocortical Lewy body disease	Braak tangle stage II	Parkinson disease (Present) [+G20] ons:57.00 dur:17.00,
CTRL3	Multiple Sclerosis and Parkinson's Tissue Bank	87	F			No abnormality detected (Present) [+NAD] ons:0.00 dur:0.00
PD8	Multiple Sclerosis and Parkinson's Tissue Bank	80	M	Parkinson's/Lewy body findings, Braak LB stage 6, Neocortical Lewy body disease	Braak tangle stage II	Parkinson disease (Present) [+G20] ons:56.00 dur:24.00
CTRL4	Multiple Sclerosis and Parkinson's Tissue Bank	81	F			No abnormality detected (Present) [+NAD] ons:0.00 dur:0.00
